# D-dimer in heart failure: Promising prognostic value but limited evidence for guiding clinical decisions

**DOI:** 10.21542/gcsp.2025.64

**Published:** 2025-12-31

**Authors:** Humberto Morais, Fidel Manuel Caceres-Loriga

**Affiliations:** 1 Hospital Militar Principal/Instituto Superior, Luanda, Angola; 2 Doctors HealthCare, Florida, USA

**To the Editor:**

We read with great interest the recent article by Halim et al., *Coagulation biomarkers as prognostic indicators in heart failure: A systematic review and meta-analysis of 14,773 patients*^1^. The authors provide a valuable synthesis of 22 studies assessing coagulation markers in heart failure (HF), identifying D-dimer as the most consistently associated with adverse outcomes. Elevated D-dimer levels were linked to higher all-cause mortality (adjusted HR 1.90; 95% CI 1.09–3.32), although wide prediction intervals (0.58–6.19) indicate substantial heterogeneity across studies.

The biological rationale for D-dimer as a prognostic marker in HF is compelling. HF involves systemic inflammation, endothelial dysfunction, and hypercoagulability; thus, elevated D-dimer may reflect ongoing fibrinolysis and disease severity. Emerging data suggest it could complement established risk models such as the MAGGIC score, offering additional prognostic information across HF phenotypes^2–4^.

However, important limitations temper enthusiasm for its clinical application. D-dimer is a nonspecific biomarker influenced by age, renal dysfunction, infection, and malignancy—factors common in HF. Most supporting evidence is observational, with heterogeneous populations, assay methods, and cut-off values, limiting standardization. Furthermore, no randomized trials have shown that D-dimer–guided therapy improves outcomes, and elevated levels likely represent systemic illness rather than a direct pathogenic mechanism^5^.

In summary, while D-dimer holds promise as a prognostic biomarker, current data do not justify its use to guide management decisions in HF. Standardized prospective studies and interventional trials are needed to define optimal thresholds and determine whether incorporating D-dimer into clinical algorithms improves risk stratification and patient outcomes.

## References

[1] Halim C, Teruna BP, Harahap IR, Fernando ET, Nugroho LBC (2025). Coagulation biomarkers as prognostic indicators in heart failure: A systematic review and meta-analysis of 14,773 patients. Global Cardiology Science and Practice.

[2] Pocock SJ, Ariti CA, McMurray JJ, Maggioni A, Køber L, Squire IB, Swedberg K, Dobson J, Poppe KK, Whalley GA, Doughty RN (2013). Meta-analysis global group in chronic heart failure, predicting survival in heart failure: a risk score based on 39 372 patients from 30 studies. Eur Heart J.

[3] Huang L, Liang L, Tian P, Zhao L, Chen Y, Huang Y, Zhou Q, Zhai M, Zhang Y, Ambrosio G, Zhang J (2022). D-dimer and outcomes in hospitalized heart failure patients across the ejection fraction phenotypes. ESC Heart Fail.

[4] Ferreira JP, Lam CSP, Anker SD, Mehra MR, van Veldhuisen DJ, Byra WM, La Police DA, Cleland JGF, Greenberg B, Zannad F (2021). Plasma D-dimer concentrations predicting stroke risk and rivaroxaban benefit in patients with heart failure and sinus rhythm: an analysis from the COMMANDER-HF trial. Eur J Heart Fail.

[5] Wu K, Van Name J, Xi L (2025). D-Dimer as biomarker for prognosis of coronary artery disease and heart failure: Reappraisal of its central role. Cardiology.

